# Timing of first focused antenatal care booking and associated factors among pregnant mothers who attend antenatal care in Central Zone, Tigray, Ethiopia

**DOI:** 10.1186/s13104-017-2938-5

**Published:** 2017-11-21

**Authors:** Gebreamlak Gidey, Birhane Hailu, Kidane Nigus, Tesfay Hailu, Woldegebriel G/her, Hadgu Gerensea

**Affiliations:** 1 Department of Midwifery, College of Health Science and Referral Hospital, Aksum University, Aksum, Ethiopia; 2 Department of Midwifery, MCHS, Adigrat University, Adigrat, Ethiopia; 3 School of Nursing, College of Health Science and Referral Hospital, Aksum University, Aksum, Ethiopia

**Keywords:** Timely anti natal care booking, Ethiopia

## Abstract

**Objective:**

Focused antenatal care became the recommended type of antenatal care following the publication of a World Health Organization trial on antenatal care where it was discovered that the traditional antenatal care approach do not necessarily improve pregnancy out-come. This study was aimed to assess timing of first focused antenatal care booking and associated factors among pregnant mothers. Facility based cross sectional study was used in the randomly selected health facilities. Total 239 pregnant women who visited antenatal clinic were selected using simple random sampling technique and data were entered and analyzed using SPSS version 20.0 software.

**Results:**

The study shows that only 41% of pregnant mothers booked timely antenatal care and the median duration of pregnancy at the first visit was 5 months. Multivariate logistic regression analysis showed that gravidity and information received on correct time of antenatal care booking from health care provider were significantly associated with timely initiation of antenatal care. Late antenatal care booking remains high in the study area and this indicated that provide information, education and communication to create community awareness is remarkable and implementing community based discussion up to the local level will be crucial.

## Introduction

Regular ANC attendance is believed to guarantee healthier pregnancies and uneventful deliveries, and women who miss visits are considered at risk of poor pregnancy outcomes [1–4].

Every day, about 1500 women across the globe die because of complications during pregnancy or childbirth, and 98% of these deaths occur in developing countries. Sub-Saharan Africa leads this death toll, accounting for 50% of all maternal deaths worldwide [1–3]. This is one of the shameful failures of world’s development. Studies on risk factors of maternal mortality have shown that lack of antenatal care increases the risk of maternal mortality [5, 6].

The number and timing of ANC visits (early booking) is an appropriate time to create awareness on signs and symptoms of pregnancy complications may lead to timely access to appropriate emergency obstetric care [4–7].

More recent Demographic and Health Survey (DHS) data illustrate that 16% of women started ANC in the first trimester in Nigeria (2008), 47% in Congo-Brazzaville (2005) and 55% in Ghana (2008) [8–13]. Moreover, DHS survey data indicate that in West Africa, 8 of 10 countries have illustrated increases, whereas, in Southern and East Africa, 6 of 11 countries have experienced declines [14].

Although there is limited evidence, late booking of antenatal care has been associated with young age, premarital status, unwanted pregnancies, high parity, and lack of formal education, low socioeconomic status, unintended pregnancy and ethnicity [10, 12–14]. While the benefits of ANC are most crucial for developing countries specially first visits by skilled providers is received by few pregnant women in those countries [15].

There are limited evidences on the time of antenatal care booking in Ethiopia which is the focus this study. Therefore, the aim of this study was to find out the prevalence of women who were booked at the recommended time and identify factors contributing for timely entry to ANC in Central Zone of Tigray regional state, Ethiopia.

## Main text

### Study area and period

This study was conducted in eight selected health centers of Central Zone of Tigray Regional State. According Tigray Regional Health Bureau 2007EFY health profile report 60.4% of births were attended by a skilled birth attendant; of which only 2.2% was clean and safe [2007EFY]. The study was conducted between January and June, 2015.

### Study design

Institutional based cross sectional study design was employed.

### Sample size

A total of 239 samples were calculated using a single population proportion formula by assuming 5% marginal error and 95% confidence interval (σ = 0.05) and prevalence of the timing of first Antenatal care booking 17% and by adding 10% of non-response rate.

### Sampling procedure

All health centers found in Central Zone of Tigray that had been giving ANC service were included. Of which using simple random sampling technique 8 health centers were selected. The sample size was distributed in proportion to average monthly load of previous year of pregnant women who made first ANC follow up at each health center by using of formula population proportion sample.

### Data collection instrument and techniques

A structured questionnaire was adopted and modified from different lecturers [9, 17, 24]. Two days training was given to all data collectors and supervisors prior to pretesting. Eight data collectors who had completed diploma in midwife were recruited.

### Data processing and analysis

Data was entered and analyzed using SPSS version 22.0. Descriptive statistics was employed to calculate frequencies and display findings. Association was measured using binary logistic regression. Based on Bivariate analysis variables that showed significant association at (p < 0.2) were entered to multivariable analysis to select Predictor variables of factors affecting timing of first ANC booking. The final model was then tested for its goodness of fit by Hosmer and Lemeshow p value and p > 0.05 was best fit. Finally, variables that showed significant association at (p < 0.05) were identified as independent predictors of time of first ANC booking.

### Ethical considerations

The Ethical approval was approved by the Institutional Review Board (IRB) of College of Health Sciences, Aksum University. Communications with the health center administrations was made through a formal letter obtained from Aksum University, College of Heath Science and TRHB. The objective and importance of the study was explained to the study participants. Data was collected after full informed written consent was obtained from participants aged 18 years and more, but age less than 18 year from the guardian. Confidentiality of the information was maintained throughout by excluding names as identification in the questionnaire and keeping their privacy during the interview by interviewing them alone.

### Socio demographic characteristics of women

Among pregnant women attending first ANC, 239 women were initiated to be included in this study. Two hundred twenty-eight (95.4%) women were responded to the interview while 11 (4.6%) did not respond. Mean age of the respondents was 28 ± 7.1 years (16–49). Majority, 152 (66.7%) of the respondents were aged 20–34 years and were mostly Tigrean of ethnic group, 223 (97.8%). Most, 156 (68.4%) had illiterate in educational background while majority, 227 (96.9%) were having no work in occupation. Only 60 (26.3%) of the respondents have greater than one thousand Ethiopian birr monthly family income (see Table 1).

**Table 1:** Socio-demographic characteristics of women who visited public health facilities in Central Zone, Tigray, Ethiopia/2015

Variables, N = 228	Frequency	Percent
Age		
15–19	22	9.6
20–34	152	66.7
35 and above	54	23.7
Occupation		
Having no work	221	96.9
Private	6	2.6
Governmental employee	1	0.4
Maternal education		
Illiterate	156	68.4
Literate	72	31.6
Husband education		
Illiterate	124	54.4
Literate	104	45.6
Marital status		
Single	4	1.8
Married	224	98.2
Religion		
Orthodox christian	224	98.2
Muslim	4	1.8
Ethnicity		
Tigrean	223	97.8
Amhara	5	2.2
Residence		
Urban	15	6.6
Rural	213	93.4
Size of the family		
≤ 4	92	40.4
≥ 5	136	59.6
Monthly income		
≤ 500	114	50
501–1000	54	23.7
≥ 1001	60	26.3
Access of ambulance service		
Yes	118	51.8
No	110	48.2

### Obstetric and reproductive history

Majority, 157 (68.9%) of the respondents were become pregnant before 19 years of age. 129 (56.6%) of participants were multiparous while the remaining 99 (43.4%) were primiparous women. Out of all respondents who gave birth before, 143 (78.6%) of them were delivered at home (see Table 2).

**Table 2:** Obstetric and reproductive history of pregnant women who visited public health facilities in Central Zone, Tigray, Ethiopia/2015

Variables, N = 228	Frequency	Percent
Age at first pregnancy (years)		
≤ 19	157	68.9
≥ 20	71	31.1
Gravidity		
Primi	99	43.4
2+	129	56.6
Have you ever give birth		
Yes	182	97.8
No	46	20.2
Place of delivery, N-182		
Home	143	78.6
Health post	7	4
Health center	27	15
Hospital	5	1.8

### Time of first ANC booking

The study finding showed that 95 (41%) of women made their first ANC visit before the fourth month of pregnancy. Whereas the majority 133 (59%) of the respondents were booked late. The pattern of ANC booking ranged from 4 to 36 weeks of pregnancy, the peak being at 20th week of pregnancy. The median duration of pregnancy at the first visit is 5 months.

### Factors affecting timely ANC booking

Bivariate and multivariate analyses were done to identify independent variables that show significant association with factors affecting timely booking for ANC for first visit. All variables which showed statistically significant association with p < 0.05 during the bivariate analysis were entered to multivariate analysis and significance was decided at p < 0.05 (see Table 3).

**Table 3:** Predictor variables of early ANC booking among mother who visited public health facilities in Central Zone, Tigray, Ethiopia/2015

Variables	Booking status of first ANC		p value	COR (95% CI)	AOR (95% CI)
	Timely booking	Late booking			
Have you ever give birth					
Yes	82 (45.1%)	100 (54.9%)	0.042	2 (1.03–4.21)	2.2 (1.07–4.67)*
No	13 (28.3%)	33 (71.7%)	++	++	
Husband/partner involvement on ANC booking timely					
Yes	75 (45.7%)	89 (54.3%)	0.048	1.9 (1.01–3.42)	1.2 (0.63–2.40)
No	20 (31.3%)	44 (68.8%)	++	++	
Rank of the mother on HDA activities					
Excellent	34 (40.5%)	50 (59.5%)		0.38 (0.24–0.59)	0.97 (0.43–0.1.18)
Good	46 (48.4%)	49 (51.6%)	0.042	2.13 (1.03–40)	1.6 (0.73–3.59)
Not ranking	15 (30.6%)	34 (69.4%)	++	++	
Ambulance services					
Yes	57 (48.3%)	61 (51.7%)	0.036	1.8 (1.04–3.02)	1.5 (0.82–2.62)
No	38 (34.5%)	72 (65.5%)	++	++	
Information received on correct time of ANC booking					
Yes	92 (45.1)	112 (54.9%)	0.006	5.8 (1.66–12.88)	4.3 (1.13–16.7)*
No	3 (12.5%)	21 (87.5%)			

* Indicates significant association

In bivariate analysis, women’s whose husband/partner involved on timely booking, who were better achievement on health developmental army, previous birth experiences and information received on accurate time of booking for antenatal care showed statistically significant association. In multivariable analysis, previous birth experiences and information received on accurate time of booking for antenatal care showed statistically significant association on timely booking for first visit (see Table 3).

As indicate in Table 3, women previous birth experiences showed strong association with timely booking for antenatal care services. Accordingly women who were information received on accurate time of booking for antenatal care were 4.3 times more likely to book timely (with in 16 week of pregnancy) for first visit of antenatal care (AOR = 4.3, 95% CI 1.13–16.70).

## Discussion

Good care during pregnancy is important for the health of the mother and the development of the fetus. Pregnancy is a crucial time to promote healthy behavior and parenting skills. World Health Organization recommends that pregnant mothers, especially those who are living in developing countries shall start ANC booking in the first 4 months of pregnancy [1]. However, in this study ninety-five (41%) respondents made ANC booking during the recommended time.

The prevalence of timely booking for antenatal care in the current study is relatively higher than the mini Ethiopia demographic health survey 2014 revealed 17% [4], 29% pregnant mothers in south Eastern Tanzania [18], 17.4% reported in South Western Nigeria [9], 27.9% pregnant mothers in Kampala Uganda [19], 35.4% of pregnant mothers in north western Ethiopia, 13.2% of pregnant mothers in Ambo, Oromia regional state Ethiopia and 26.2% of pregnant mothers in Debrebrhan town, Amhara, Ethiopia were booked timely for antenatal care respectively [20, 22, 24].

This study result is also relatively lower compared to the findings of other studies which reported 80% in Indonesia [16], and 51.8% in Ethiopia [21], Such a relatively high difference in prevalence of timely booking in antenatal care is largely due to variation in year of study, socio demographic features of the study participants, media of information among health care givers, knowledge of mothers on importance of early ANC booking and poor community awareness on the issue of focused antenatal care.

In this study socio-demographic factors like age of the mother, educational background of the mother, marital status, income, religion, ethnicity, husband involvement and residence were not found to be related to timely Antenatal care booking. In contrast of this result, a study from Tanzania confirmed that not being supported by the husband or partner was identified as factors associated with a later antenatal care booking [18]. Similarly studies conducted in Ghana, Nigeria, Kenya, Malawi, Oromia and SNNP of regional sates in Ethiopia reveled that, age, maternal education, older multifarious pregnant women are at particular predictors for antenatal care booking and unmarried younger women are also risk factor for booking early than married women [10, 18, 19, 22]. This difference could be due to sample size, time of study. This might be due to women in the older age group are more likely to have many children to care and many of the older pregnant women’s might have ingrained cultural biases against formal health care.

Birth experience in this study was associated with early booking for antenatal care. It is similar with the study done in Gedeo zone, SNNP region, Ethiopia during 2014 and parity was found as significant factors that influence timing of first Antenatal care booking [23]. This study is also consistent with the study done in Adigrat, Tigray, Ethiopia that showed that pregnant women who had parity one and above decreased the likelihood of late booking than the reference category [21].

Similarly this study is also consistent with the study done in Zambia and in United Kingdom which was showed that multiparous women were more likely to initiate ANC early compared to primiparous women [17, 26].

Regarding awareness about FANC, the study revealed that women who were informed on the correct time of booking were more likely to initiate ANC early compared to those without. This finding is similar to what a study done in Ambo town and other studies in Oromia, Gedeo zone, SNNP Ethiopia found out in their study where women who were well informed about timely ANC were more likely to book for ANC within the recommended time [22–25]. This similarity could be due the application of the national health policy of focused antenatal care in the nation. Furthermore, this study was able to confirm that pregnant women who had not have information on benefits of timely booking for ANC are derived from staring early, tend to start ANC late.

## Conclusion and recommendation

Early antenatal care attendance remains low in both rural and semi-urban districts indicating that the importance of early initiation in Central Zone of Tigray regional state, Ethiopia is yet to be appreciated. Multiparty and receiving correct information on time of antenatal care booking have an association with early ANC booking. In order to improve this concern, it is important to provide information, education and communication to create community awareness regarding the time of first antenatal booking with its advantages. Similarly Regional Health Bureau and Ministry of Health should formulate a strategy to maximize the number of women’s who have information on it further community study should be recommended to explore additional causes for late antenatal booking.

## Limitation

Data from health facilities are potentially useful for monitoring time ANC in the number of pregnant mothers but have severe limitations. Analysis was based on routinely collected ANC data from public health institution. There is a possibility of both under and over reporting of timely ANC. Since cross sectional study does not show cause and effect relationship.

## Abbreviations

CI: confidence interval
AOR: adjusted odd ratio
ANC: anti natal care
SPSS: Statistical Package for Social Sciences
SNNP: Southern Nation and Nationalities of People
HDA: Health Development Army
EDHS: Ethiopian Demographic and Health Survey
TRHB: Tigray Regional Health Bureau
